# A Mask-Shaped Respiration Sensor Using Triboelectricity and a Machine Learning Approach toward Smart Sleep Monitoring Systems

**DOI:** 10.3390/polym14173549

**Published:** 2022-08-29

**Authors:** Jonghyeon Yun, Jihyeon Park, Suna Jeong, Deokgi Hong, Daewon Kim

**Affiliations:** 1 Department of Electronics and Information Convergence Engineering, Institute for Wearable Convergence Electronics, Kyung Hee University, 1732 Deogyeong-daero, Yongin 17104, Korea; 2 Institute for Wearable Convergence Electronics, Kyung Hee University, 1732 Deogyeong-daero, Yongin 17104, Korea; 3 Department of Occupational Therapy, College of Medicine, Wonkwang University, 460 Iksan-daero, Iksan 54538, Korea; 4 Department of Electronic Engineering, Institute for Wearable Convergence Electronics, Kyung Hee University, 1732 Deogyeon-daero, Yongin 17104, Korea

**Keywords:** triboelectric nanogenerator, smart electronics, k-mean clustering, sleep monitoring system

## Abstract

Daily sleep monitoring is limited by the needs for specialized equipment and experts. This study combines a mask-shaped triboelectric nanogenerator (M-TENG) and machine learning for facile daily sleep monitoring without the specialized equipment or experts. The fabricated M-TENG demonstrates its excellent ability to detect respiration, even distinguishing oral and nasal breath. To increase the pressure sensitivity of the M-TENG, the reactive ion etching is conducted with different tilted angles. By investigating each surface morphology of the polytetrafluoroethylene films according to the reactive ion etching with different tilted angles, the tilted angle is optimized with the angle of 60° and the pressure sensitivity is increased by 5.8 times. The M-TENG can also detect changes in the angle of head and snoring. Various sleep stages can be classified by their distinctive electrical outputs, with the aid of a machine learning approach. As a result, a high averaged-classification accuracy of 87.17% is achieved for each sleep stage. Experimental results demonstrate that the proposed combination can be utilized to monitor the sleep stage in order to provide an aid for self-awareness of sleep disorders. Considering these results, the M-TENG and machine learning approach is expected to be utilized as a smart sleep monitoring system in near future.

## 1. Introduction

Human beings sleep at least a quarter of their lifetime. During sleep, learned knowledge is stored in the brain, damaged muscles recover, and cognitive ability is maintained, simultaneously [1,2,3]. Hence, the processes of learning, recovery, and cognition can be disturbed by sleep deprivation resulting from sleep disorders. More seriously, sleep disorders can induce various diseases, including hypertension, coronary disease, diabetes, and cardiac ischemia [4,5]. In addition, sleep deprivation could reduce cognitive ability, increasing the possibility of terrible accidents, such as car accidents. However, despite the risks associated with sleep disorders, only a few patients are diagnosed and cured due to a lack of a self-recognition of sleep disorders [6]. To recognize and cure sleep disorders, investigating sleep stages is important. Polysomnography is a standard test to evaluate sleep stages and wakefulness. This test can record various physiological functions that change during sleep at the same time through brain waves, electromyography, and breathing patterns [7]. The sleep stages are composed of rapid eye movement (REM), non-REM 1 (N1), non-REM 2 (N2), and non-REM 3 (N3), respectively [8,9,10,11]. Each sleep stage can be classified based on the changes generated in brain waves during sleep [12,13]. However, conventional clinical sleep monitoring systems are notably limited for daily life applications. They typically require specialized equipment in clinical places, experts to conduct sleep monitoring, and high cost. Since the characterization of sleep is mainly based on electroencephalogram (EEG) analysis [7], alternative sleep monitoring systems without these limitations are highly desired for daily life applications.

One attractive alternative sleep monitoring system is using a respiration monitoring device, which can be utilized during sleep. The patterns of respiration reflect the degree of the activation of the parasympathetic nervous system, which changes according to sleep stages [14,15,16,17,18]. During non-REM sleep, the parasympathetic nervous system tension increases and energy consumption decreases, resulting in a decrease in respiration rate. On the other hand, during REM sleep, breathing patterns and heartbeats are irregular. Irregular breathing pattern changes during REM sleep and changes in upper airway muscle tone may exacerbate sleep apnea [19]. Therefore, sleep disorders, such as sleep apnea, can be easily detected by respiration monitoring during sleep [20,21]. Previous studies have classified the sleep phase through breathing signals, therefore, respiration sensors can play a key role in confirming the changes in respiration patterns during sleep [22]. These respiration sensors should be capable of maintaining a long-time operation because respiration monitoring during sleeping is conducted throughout the night. Conventional respiration sensors have depended on external power sources, such as batteries, to sustain a long-time operation. However, these batteries possess fatal problems, such as their bulky size, limited service time, and need for frequent replacement and careful disposal to avoid pollution. Hence, battery free respiration sensors are highly required in respiration monitoring during sleep.

Respiration generates a mechanical energy based on the air flow, which is induced by the pressure difference between inside and outside of the body, semi-permanently. By scavenging this mechanical energy, the battery free respiration sensors can be implemented. Recently, various energy harvesters have been developed which convert the mechanical energy into the electricity, such as a piezoelectric nanogenerator (PENG) [23,24,25], triboelectric nanogenerator (TENG) [26,27], and electromagnetic generator (EMG) [28,29]. Among them, the TENGs are the most attractive generator to implement the battery free respiration sensors because of the TENGs ability to scavenge the respiration-induced mechanical energy. A TENG can generate the electrical energy from the mechanical energy based on a coupling effect between contact electrification and electrostatic induction [30,31,32,33,34,35,36]. The electrical output generated from the TENG dominantly relies on the displacement between two materials, surface charge density, and contact surface, respectively [37,38]. Hence, the TENG can harvest mechanical energies with a low frequency. This indicates that the TENG can effectively harvest the respiration-induced mechanical energy with the frequency range of 0.2 to 0.3 Hz, which is typical frequency range of respiration. TENGs possess a wide spectrum of feasible materials which can be utilized as both the triboelectric dielectric layers or electrodes in the TENGs [39,40,41,42]. For example, pollution-free materials, such as polymers, can be utilized to fabricate the TENG-based battery free respiration sensor. Based on these results, various self-powered sensors have been proposed in previous research.

Considering these advantages, a TENG can be a promising candidate for application in a battery free sensor. Several TENG-based sensors have been developed to conduct sleep monitoring [21,43,44,45,46,47,48,49,50,51]. For example, the e-textile was attached on the stomach for sleep monitoring [21]. The e-skin also was utilized for sleep monitoring [43]. For monitoring sleep, TENG-based large scale pads were employed [44,45,46]. Flexible material based TENGs were also used to conduct sleep monitoring [47,48,49,50,51]. With these proposed TENG-based sleep monitoring systems, sleep disorders can be easily detected, such as apnea and hypopnea, because they dramatically change respiration patterns. However, sleep disorders, such as hypersomnia and parasomnias, cannot be detected with these systems. To recognize these types of sleep disorders, all sleep stages need to be investigated. Then, an entire sleep can be precisely evaluated and various sleep disorders can be detected by confirming the sleep stages. Hence, in order to recognize various sleep disorders, the TENG-based sensor should be capable of classifying sleep stages.

Patterns of respiration during sleep can be utilized to classify the sleep stages with the TENG-based respiration sensors, because the respiration of a normal person characteristically changes according to the particular sleep stages. With a TENG-based respiration sensor, the pattern of generated electrical signals can be detected, which correspond to the respiration patterns of sleep stages. These electrical signals can be classified with the assistance of machine learning based pattern recognition. This is because machine learning can study and classify the associated features among the arbitrary data by correlating the data with similar characteristics [52,53]. After learning, arbitrary data can be classified using the pre-learned results of the machine learning. Hence, combination of the TENG-based respiration sensors and machine learning can be highly effective to classify the sleep stage.

Herein, the combination of the mask-shaped triboelectric nanogenerator (M-TENG) and machine learning was demonstrated for a sleep monitoring system. The fabricated M-TENG was composed of the polytetrafluoroethylene (PTFE) film, aluminum (Al) tape, and modified acrylonitrile butadiene styrene (ABS-A100). The fabricated M-TENG was lightweight and pollution-free because of its components. The M-TENG demonstrated excellent ability as a respiration sensor by successfully distinguishing the oral and nasal breath. To increase the sensitivity of the M-TENG, the reactive ion etching (RIE) was conducted on the surface of the PTFE film. Then, the brain waves and respirations were simultaneously investigated using a dry sensor interface-24 (DSI-24) and M-TENG during sleep. As a result, changes in the respiration patterns were observed according to sleep stages.

To classify sleep stages using respiration patterns, the k-mean clustering method was adopted because k-mean clustering can classify arbitrary data into groups with the similar characteristics [54,55,56,57,58]. To increase classification accuracy, the output voltage generated from the M-TENG was converted into a relative value. This can suppress the variation of the output voltage. The weight value for the respiration rate was also investigated, and the highest classification accuracy of 96.67% was recorded at the weight value of 0.1. Based on these results, the centroids of each sleep stage were optimized. With these centroids, the high classification accuracy of 89.17%, 86.67%, 88.33%, 87.50%, and 84.17% were recorded at the stages of awake, N1, N2, N3, and REM, respectively. These results indicate that the proposed system can help to recognize sleep disorders using classified sleep stages. Considering these results, collaboration of the M-TENG and machine learning is expected to be utilized as the promising smart sleep monitoring systems in the near future.

## 2. Materials and Methods

### 2.1. Fabrication of the Mask-Shaped Triboelectric Nanogenerator (M-TENG)

The substrate of the M-TENG was designed using a Fusion 360 (Autodesk, SAN Rafael, CA, USA) and printed by a 3D-printer (CUBICON, Seongnam, Korea) with the ABS-A100 filament because of the robustness and stiffness of the acrylonitrile butadiene styrene (ABS-A100) filament (CUBICON, Seongnam, Korea). Then, a reactive ion etching process was performed at 30 sccm, 30 W for 180 s on the surface of the commercial polytetrafluoroethylene film (2 cm × 4 cm with thickness of 100 μm, ALPHAFLON, Seoul, Korea). Then, the PTFE film was attached on the ABS-A100 substrate as the active layer inducing the triboelectrification with the aid of the double-sided tape. Aluminum tape possessing only one side of the conductivity was utilized as the electrode.

### 2.2. Working Mechanism of the M-TENG

**Figure 1:**
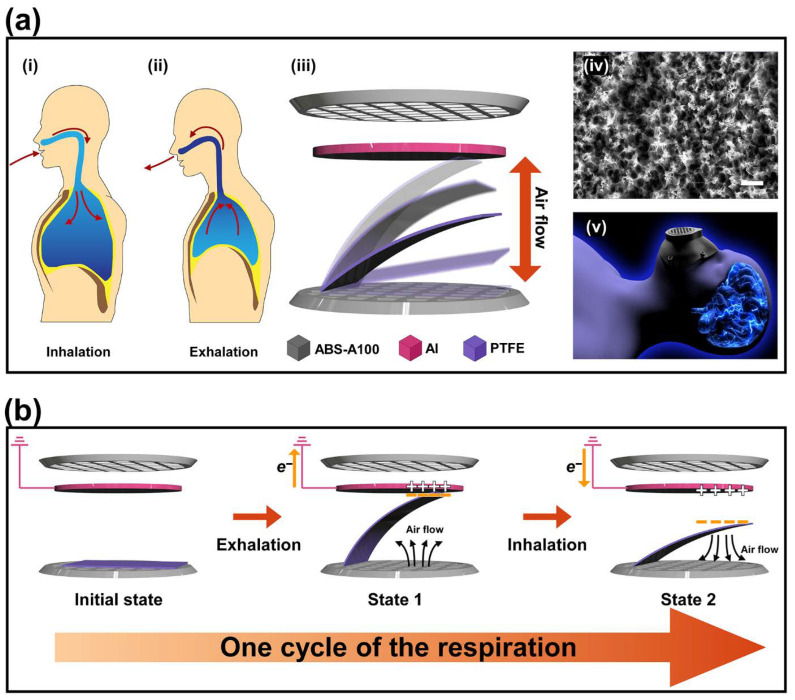
The schematic illustration of the proposed M-TENG and its mechanism to generate the triboelectricity. (**a**), Diagram of (**i**) inhalation and (**ii**) exhalation. (**iii**) Schematic illustration of the proposed M-TENG. (**iv**) Surface morphology of the utilized PTFE film with the scale bar of 1 μm. (**v**) Utilization of the M-TENG. (**b**) The mechanism of generating the electricity with the M-TENG during one cycle of respiration.

The diagram of the fabricated mask-shaped triboelectric nanogenerator (M-TENG) and its working mechanism are illustrated, respectively, as shown in Figure 1. In the respiration rest state, an intrapulmonary and intrapleural pressure of 760 mmHg are observed, which are corresponding to atmospheric pressure. During inhalation, the intrapulmonary and intrapleural pressure decrease to 757 mmHg and 754 mmHg as the diaphragm descends. This pressure difference in lungs induces air flow from the outside into the lungs (Figure 1a(i)). Then, the intrapulmonary and intrapleural pressure increase with the pressure of 763 mmHg and 757 mmHg as the diaphragm is raised during exhalation, respectively. As a result, the air in the lungs is expelled to the outside, as shown in Figure 1a(ii). Considering this, the M-TENG was designed to generate triboelectricity based on the respiration-induced air flow. The mask frame of the M-TENG was sealed to prevent the leakage of the air flow. The Figure 1a(iii) shows the schematic illustrations of the proposed M-TENG operated in a single-electrode mode. The fabricated M-TENG was composed of the modified acrylonitrile butadiene styrene (ABS-A100) as a mask frame, Al tape as an electrode, and polytetrafluoroethylene (PTFE) as the electrostatic inducing material, respectively. The PTFE film was attached to the mask frame of the M-TENG. Additionally, the reactive ion etching process was conducted on the surface of the PTFE film and the nanostructures were formed on the surface of the PTFE film after the RIE process, as shown in Figure 1a(iv). An illustration of wearing the M-TENG is displayed in Figure 1a(v).

The working mechanism of the proposed M-TENG is described, as shown in Figure 1b. At the initial state, the PTFE film is placed on the bottom side. When the air flow is created by exhalation, the attached PTFE film is raised and approaches the Al electrode. When the surface of the PTFE is in contact with the Al electrode, the surface of the PTFE film is easily charged with negative charges, as shown in state 1. This is because the PTFE, which contains a rich amount of 6 fluorine in its polymer chain, possesses high electronegativity. At the same time, positive charges appear on the surface of the Al electrode to maintain the electrical equilibrium. When the surface of the Al electrode is filled with positive charges, the electrons are pushed away from the Al to the ground. This is because the electrons in the Al electrode can be easily moved compared to the negative charges on the PTFE film. This movement of electrons in the Al electrode generates a current in the direction from ground to Al electrode. When inhalation occurs, the opposite direction of air flow is generated and the PTFE film begins to fall. As a result, the effect of the negative charges on the Al electrode decreases, and the electrical equilibrium is broken again. To balance the electrical equilibrium, electrons are pumped from the ground to the Al electrode, as shown in state 2. Finally, the current generated from the M-TENG during inhalation flows with the opposite direction than the current produced from the M-TENG during exhalation.

### 2.3. Characterization

After the reactive ion etching process, the surface morphology of the PTFE was investigated using a high-resolution field emission scanning electron microscope (MERLIN, Carl Zeiss, Jena, Germany). The surface morphology and roughness of the PTFE film was investigated by atomic force microscopy (XE7, Park Systems, Suwon, Korea).

### 2.4. Measurement Setups

To measure the basic output characteristics of the M-TENG (open-circuit voltage and short-circuit current), an electrometer (Keithley 6514, Tektronix, Beaverton, OR, USA) was utilized. To measure the respiration during sleeping, the Arduino board was utilized with 1 GΩ of the serially connected resistance. For measuring the EEG, the dry electrode EEG headset (DSI-24, WEARABLE Sensing, San Diego, CA, USA) was utilized. For accurate EEG measurement, the signal was intensively analyzed from the part where the specific EEG signal was mainly emitted.

### 2.5. Human Subject Study

The experiment was conducted using human subjects in compliance with all the ethical regulations under a protocol (ID: WKIRB-202110-HR-081) approved by the Institutional Review Board at University of Wonkwang, Republic of Korea. Additionally, the experiments were performed according to the actual biorhythm of each participant. The conditions of the experiment were a temperature of 25 °C with a relative humidity of 50%. Furthermore, the home of each participant was selected as a measurement place where participants could feel comfortable. During sleep, light and sound, which can stimulate the brain of the participant, were strictly controlled. The experiments were conducted with participants A, B, and C, with an age in their twenties, and all participants breathed with their nose. From two weeks ago, performing the experiment, all participants slept regularly in order to prevent the delayed sleep phase syndrome.

## 3. Experimental Results

### 3.1. Characteristics and Electrical Output of the Fabricated M-TENG

**Figure 2:**
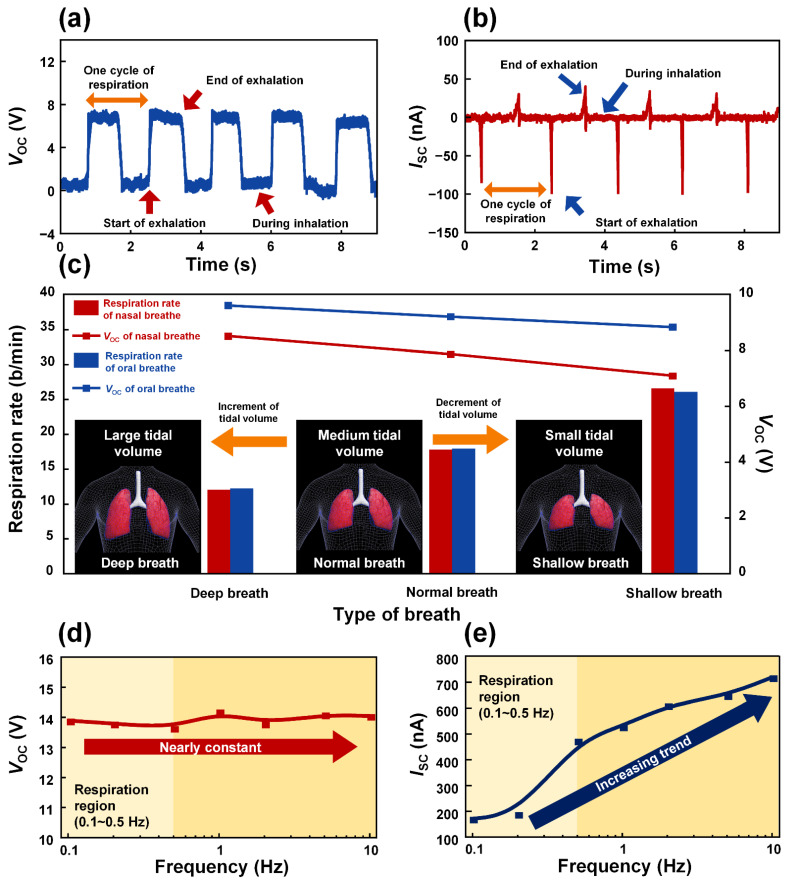
The electrical outputs generated by the M-TENG. (**a**) The *V*_OC_ and (**b**) *I*_SC_ generated by the respiration. (**c**) The respiration rates and *V*_OC_s acquired from the M-TENG for deep, normal, and shallow breathing, respectively. The (**d**) *V*_OC_s and (**e**) *I*_SC_s generated by the M-TENG according to the frequency.

To verify the working mechanism of the M-TENG, the electrical output generated from the M-TENG was investigated. A cycle of the respiration can be defined as a repetition of inhalation and exhalation. When one breathing cycle was conducted through the nose, the open-circuit voltage (*V*_OC_) of 7.89 V of and short-circuit current (*I*_SC_) of 82 nA were generated from the M-TENG, respectively, as shown in Figure 2a,b. In the rest state of the respiration (initial state), the attached PTFE film was located on the ABS-A100 substrate and maintained electrical equilibrium. When exhalation was conducted, the attached PTFE film approached the Al electrode and the *V*_OC_ of 7.89 V was generated (state 1). When the PTFE film was in contact with the Al electrode, the amplitude of the electrical output was constant because the electrical equilibrium was maintained during the exhalation. When the state of respiration was converted from exhalation to inhalation, the PTFE film began to fall. Then, the electrical equilibrium was broken and the value of the *V*_OC_ decreased (state 2). This is because the effect of negative charges on the Al decreased when the PTFE film fell during inhalation. These results successfully demonstrate the proposed working mechanism of the M-TENG described in Figure 1b.

The weight of the PTFE film is also considered a key factor in generation of triboelectricity with the M-TENG. For example, if the attached PTFE film is lightweight, the PTFE film will remain to be attached at the Al electrode due to the strong attraction force induced by the triboelectrification between PTFE film and Al electrode. When the PTFE film is attached to the electrode, the M-TENG cannot detect respiration. On the other hand, if the weight of the attached PTFE film is heavy, the PTFE film cannot be moved by the respiration-induced air flow. Accordingly, the weight of the attached PTFE film should be optimized to detect respiration with the high sensitivity. To confirm the optimized weight of the attached PTFE film, several M-TENGs with various weights of the PTFE films were fabricated. Then, each electrical output generated from the fabricated M-TENGs was investigated, as shown in Figure S1. As a result, the highest *V*_OC_ of 7.89 V and *I*_SC_ of 82 nA were generated from the M-TENG, with an attached 1.5 g PTFE film. Furthermore, voltage was chosen as the signal for monitoring the respiration because the value of the generated voltage was large despite the small contact area between the PTFE film and Al electrode.

The volume and intensity of air during respiration can vary depending on individual characteristics, such as respiration method (mouth or nose) and size of the lungs. The fabricated M-TENG needs to be able to detect these various parameters related to breathing in order to accurately observe respiration during sleep. Considering this, the electrical outputs generated from the M-TENG with oral and nasal breathing were investigated by analyzing the tidal volume of the respiration, as shown in Figure 2c. When a shallow breath was performed, the smallest tidal volume was observed. This smallest tidal volume induced the weakest air flow, resulting in the decrement in the raised height of the PTFE film. Then, the generated electrical output was lowered compared to that of a normal breath. The highest tidal volume was observed during a deep breath. The high tidal volume of the deep breath meant the attached PTFE film at the M-TENG was in contact with the Al electrode with the strongest contact force. Hence, the highest electrical output can be generated from the M-TENG by a deep breath. The *V*_OC_s of 8.54 V, 7.89 V, and 7.12 V were measured with shallow, normal, and deep breath through the nose, respectively. The M-TENG generated *V*_OC_s of 9.64 V, 9.24 V, and 8.87 V with the shallow, normal, and deep breathing through the mouth, respectively. Based on these results, the differences between respiration patterns, such as the oral or nasal breath and deep, normal, and shallow breath, can be accurately detected with the fabricated M-TENG. In Figure 2d,e, the *V*_OC_s and *I*_SC_s generated from the M-TENG were investigated by varying the working frequency. The range of working frequency was 0.1 Hz to 10 Hz, which was including the frequency of respiration (0.1 Hz to 0.5 Hz). Based on these results, the proposed M-TENG can be utilized to detect the various respiration with different respiration rate. Additionally, when the frequency was increased, the M-TENG generated a constant voltage, which was approximately 13.93 V. In contrast, the *I*_SC_ increased as the frequency increased.

**Figure 3:**
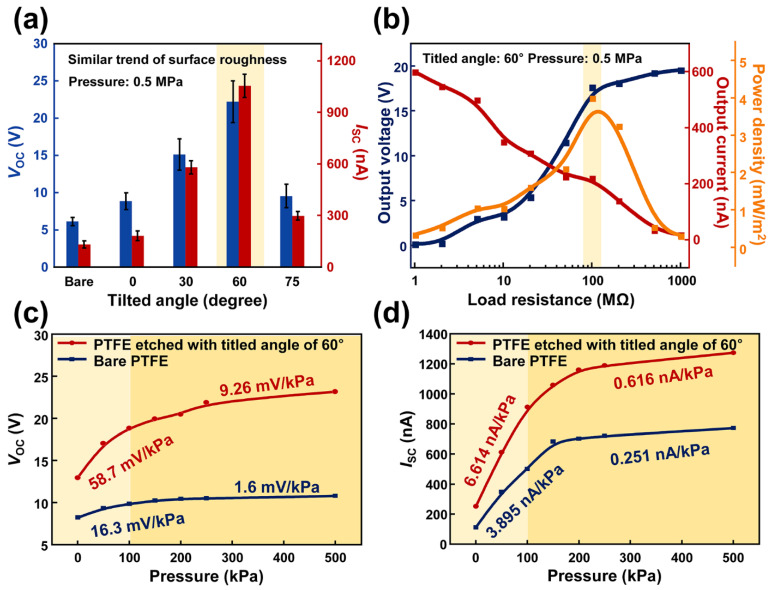
(**a**) The electrical output generated from the M-TENG according to the tilted angle. (**b**) Electrical power density of the M-TENG with various load resistances. The (**c**) *V*_OC_ and (**d**) *I*_SC_ generated from the M-TENGs with bare PTFE film and PTFE film etched with tilted angle of 60°.

When the air pressure of 0.5 MPa was applied to each M-TENG, the highest electrical output was produced from the M-TENG with the etched PTFE film, which was etched with the tilted angle of 60°, as shown in Figure 3a. This M-TENG device can generate the power density of 4.03 mW/m^2^, with a load resistance of 100 MΩ, as shown in Figure 3b. The improved pressure sensitivity was demonstrated as shown in Figure 3c,d by comparing the electrical output generated from the M-TENG with bare PTFE film and PTFE film, which was etched with the tilted angle of 60°. The range of applied air pressure was 0.133 kPa to 500 kPa. In an air pressure range of 0.133 kPa to 100 kPa, the slope of *V*_OC_ and *I*_SC_ generated from M-TENG with PTFE film etched with tilted angle of 60° recorded 3.6- and 1.7-times increased value compared to *V*_OC_ and *I*_SC_ from M-TENG with bare PTFE film. Additionally, in the air pressure range of 150 kPa to 500 kPa, 5.8- and 2.5-times of increment were observed. This is because the tilted RIE process can create an oblique morphology on the surface of the PTFE, which can increase the effective contact area [59]. Considering these results, the M-TENG with PTFE film, which was etched with the tilted angle of 60°, is an optimized device for detecting respiration with the highest-pressure sensitivity. Furthermore, the voltage was selected as the signal to detect the respiration. The current generated from the M-TENG is affected by the working frequency (Figure 2e) and pressure. In contrast, the amplitude of voltage generated from the M-TENG is dependent on pressure and the working frequency can rarely affect the voltage generated from the M-TENG. Therefore, by using a voltage as the signal, the M-TENG can distinguish the respiration with different frequency and pressure, respectively.

### 3.2. Electrical Signal Collection According to the Sleep Stage

**Figure 4:**
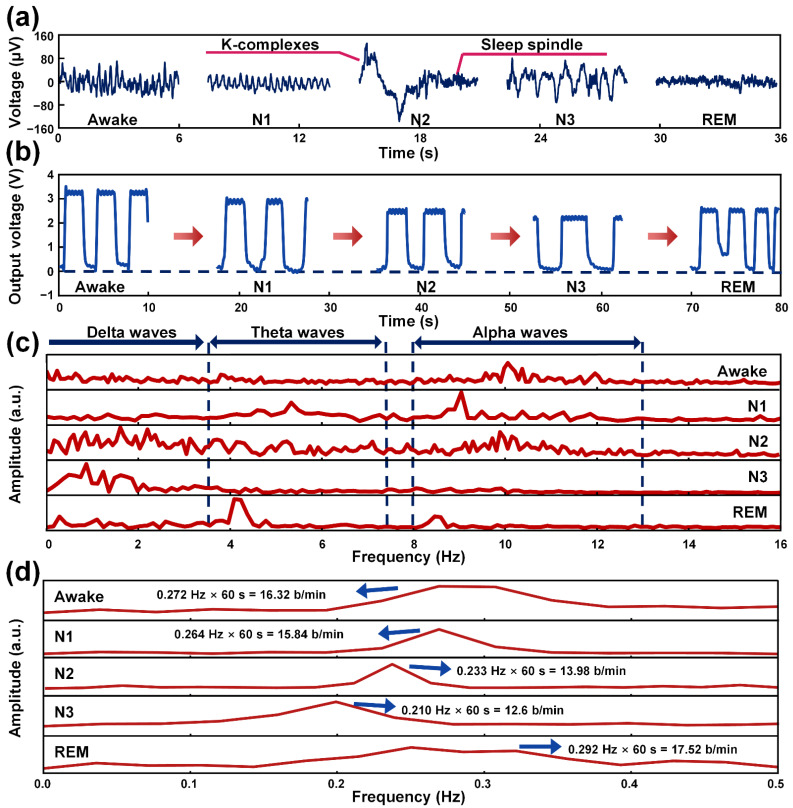
The electroencephalogram of the brain acquired by the DSI-24 and electrical output generated from the M-TENG according to the sleep stage. (**a**) The brain waves of the awake, N1, N2, N3, and REM stages. (**b**) The electrical outputs generated from the M-TENG for each sleep stage. (**c**) The amplitudes of the brain wave for each sleep stage in the frequency domain. (**d**) The amplitudes of electrical outputs for the awake, N1, N2, N3, and REM stage in the frequency domain after fast Fourier transform.

During sleep, brain waves change depending on sleep stages. To confirm the changes in brain waves, electroencephalograms (EEG) were recorded through a dry sensor interface-24 (DSI-24). A potential difference occurs between the outside and the inside of the brain because current is generated by the ion movements of the nerve cells in the brain when the nerve cells are stimulated and activated. This potential difference can be successfully recognized by attaching the electrodes of the DSI-24 to the scalp. The changes in the brain waves and electrical output generated from the M-TENG according to the sleep stage can be detected, as shown in Figure 4a,b. The potential differences generated from the brain were systematically analyzed in the time and frequency domain, respectively, as shown in Figure 4a,c. Sleep and breathing are closely related. During non-REM sleep, the respiratory rate and the heart rate become regular and constant. On the other hand, during REM sleep, breathing patterns and heartbeats show irregular patterns. In particular, it causes the most notably anomalous breathing pattern during phase-phase REM sleep. The metabolic control of respiration is also altered during sleep. The hypoxic ventilatory drive decreases during non-REM sleep and additionally decreases during REM sleep. Hypercapnic ventilatory response also decreases during non-REM sleep and is completely lost during REM sleep [60].

To sleep, people close their eyes and maintain a comfortable physical and mental state, without being excited by external stimuli. In this awake stage, alpha waves with a frequency range of 8 to 13 Hz are predominantly generated in the occipital lobe of the brain. Considering this, the potential difference of the occipital lobe was investigated and analyzed to confirm the alpha waves. As shown in Figure S2, O1 and O2 electrodes of the DSI-24 were located at the near part of the occipital lobe. Hence, the acquired potential differences from the O1 and O2 electrodes were selected and analyzed to confirm the alpha waves. As a result, the alpha waves were detected, as shown in Figure 4a,c. In the N1 stage, the amplitude of the alpha waves began to decline and theta waves emerged, simultaneously. The amplitude of the potential difference in the brain also decreased. The frequency range of theta waves is 3.5 to 7.5 Hz, and they are mainly generated from the side of the brain. To observe theta waves, electrodes of the DSI-24, which were attached on the nearby frontal lobe (F7 and F8), temporal lobe (T3, T4, and T5), and parietal lobe (P6), were utilized. When the sleep stage was changed from the N1 to the N2 stage, delta waves were detected at a frequency below 3.5 Hz, as shown in Figure 4c. Compared to the awake and N1 stage, the N2 stage cannot be confirmed with the specific brain waves. However, k-complexes and sleep spindles were observed in the N2 stage, as shown in Figure 4a. These k-complexes and sleep spindles phenomena are only observed in the N2 stage. Therefore, the observations of the k-complexes and sleep spindles are strong evidence to confirm the N2 stage of sleep. In the N3 stage, the deepest sleep occurs, called slow wave sleep, and delta waves were predominantly generated in this stage, as shown in Figure 4c. In addition, the highest averaged amplitude of the potential difference was observed among sleep stages, as shown in Figure 4a. Finally, in the REM stage, the lightest sleep occurs and the condition of the body becomes unstable. Theta waves are predominantly generated in this stage, as shown in Figure 4c. Based on these results, each sleep stage can be classified based on the EEG data.

The sleep stage can be classified as REM stage and non-REM stage (N1, N2, and N3) based on biological activity during sleep. During the REM stage, the human body enters a physiologically unstable state, which includes the appearance of the eye movement despite muscle relaxation. Moreover, in the REM stage, the sympathetic nervous system is activated, which tends to increase the respiration rate and tidal volume [19]. This unstable state can also affect respiration patterns, inducing irregular respiration during the REM stage. Therefore, biological behavior, such as respiration and muscle movements, become unstable in order to be converted to the active state (awake) from the sleep state. On the other hand, during non-REM sleep (N1, N2, and N3), the tidal volume and speed of respiration decrease, and respirations become regular due to the activated parasympathetic nervous system. The parasympathetic nervous system is more highly activated during sleep than when quietly resting. When the parasympathetic nervous system is activated, the body begins to relax, and the rate and intensity of respiration are decreased. Hence, the electrical output generated from the M-TENG during non-REM sleep should be lower than that in the awake state because the movement of the attached PTFE film in the M-TENG will be reduced due to the weakened air flow. In addition, compared to the N1, N2, and N3 stage, the respiration rate and output voltage measured by the M-TENG in the REM stage should reflect a difference in respiration pattern. Considering this, the electrical signal generated from the M-TENG with respect to respiration was investigated according to the sleep stages, in terms of the time domain, as shown in Figure 4b. For participant A, the output voltage with each sleep stage can be checked at Table S1. Compared to the awake state, the generated electrical outputs during non-REM sleep (N1, N2, and N3 stages) decreased as the parasympathetic nervous system activated, respectively. Furthermore, the amplitude of the generated electrical signal in the REM stage showed irregular patterns due to uneven respiration in the REM stage. These results successfully demonstrate changes in the electrical output generated from the M-TENG according to the sleep stage. The sampling rate was 0.017 s and the length of each data was approximately 1800 with a measurement time of 30 s for the M-TENG. The measurement was continually conducted with this sampling rate and sampling time until the participant woke up.

To acquire information related to the frequency of respiration during sleep, the fast Fourier transform (FFT) was conducted with the electrical outputs generated from the M-TENG by the respiration at each stage of sleep. The frequencies of 0.272 Hz, 0.264 Hz, 0.233 Hz, 0.210 Hz, and 0.292 Hz were acquired after conducting FFT analysis. When the acquired frequencies were converted to the respiration rate (breath/minute), the respiration rates of 16.32 b/min, 15.84 b/min, 13.98 b/min, 12.6 b/min, and 17.52 b/min were calculated at sleep stages of awake, N1, N2, N3, and REM, respectively, as shown in Figure 4d. The respiration rate (number of breaths per minute) was employed because the respiration rate can show the characteristics of respiration in a specific period rather than one single breath. Compared to sleep stages of awake, N1, N2, and N3 stage, the respiration rate of the REM stage showed a wide bandwidth in the frequency domain indicating that irregular respiration was conducted during the REM stage. Furthermore, the decreased trend of the respiration rates of the non-REM sleep states (N1, N2, and N3) were observed compared to that of the awake state. Considering these results, the respiration rates of each sleep stage can also be utilized as standards to classify the sleep stage.

**Figure 5:**
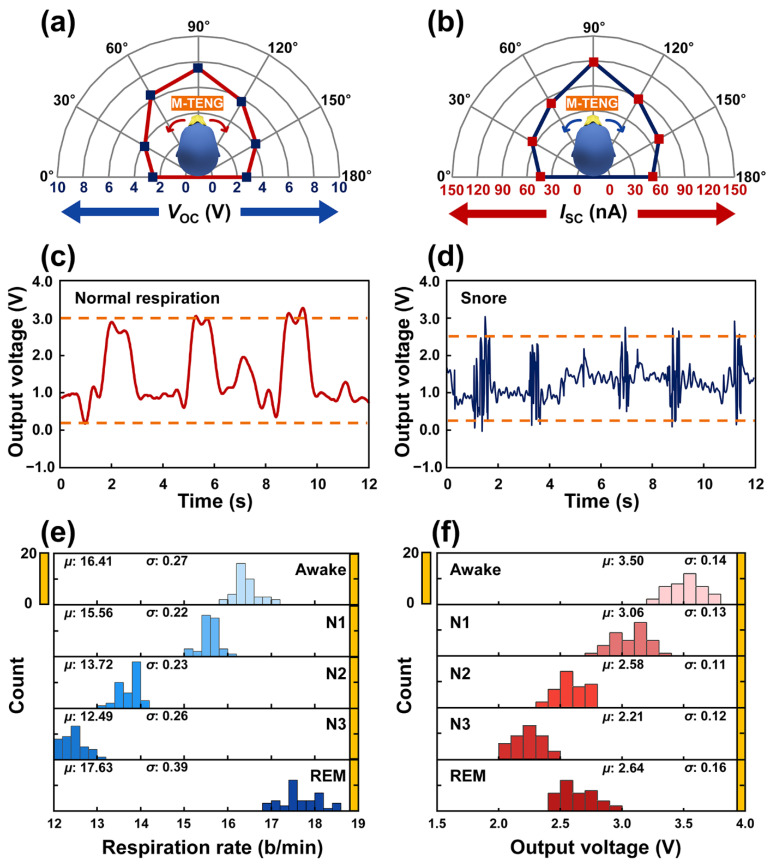
The potential of the M-TENG as the respiration sensor and the measurement result of the respiration during sleeping with the M-TENG. (**a**) The open-circuit voltage generated from the M-TENG according to the angle of the head. (**b**) The short-circuit current generated from the M-TENG according to the angle of the head. (**c**) The electrical output generated from the M-TENG during normal respiration. (**d**) The electrical output generated from the M-TENG during snoring. (**e**) The distributions of the respiration rates and (**f**) the measured output voltages with the respiration of participant A.

To confirm the potential of the M-TENG as a respiration sensor during sleep, the electrical outputs generated from the M-TENG were investigated according to the angle of the head during sleep, as shown in Figure 5a,b. When the head of the participant was turned to the left during sleep, the angle of 0° was assigned. Hence, the angles of 90° and 180° implied that the participant slept looking at the ceiling and with the head turned to the right, respectively. The highest electrical outputs were observed at an angle of 90°. This is because the angle enhances the separation process of the PFE film during inhalation. When the angle of the head was out of 90°, the air flow required for the separation process of the PTFE film during inhalation increased. Hence, the proposed M-TENG can be used to detect the sleeper’s head position by comparing the electrical output generated from the M-TENG for each head angle during sleep. In addition, snoring can be detected with the M-TENG. A continuity was observed at the shape of the electrical output generated from the M-TENG during normal respiration, as shown in Figure 5c. However, when a person snores while sleeping, respiration occurs discretely rather than continuously. In this case, the shape of the electrical output generated from the M-TENG exhibited a vibrating shape, as shown in Figure 5d. Furthermore, the amplitude of the electrical signal acquired in the snore state decreased compared to the electrical signal acquired during normal respiration, due to reduced tidal volume. Since the M-TENG can detect breathing with a high frequency, as shown in Figure 2d,e, the M-TENG can detect the electrical signal with high frequency, such as the signal generated from snoring. Considering these results, the proposed M-TENG can be utilized as a respiration sensor during sleep.

The electrical outputs and respiration rates acquired from the M-TENG were collected and systematically analyzed according to sleep stage. In Figure 5e,f, the distribution of the respiration rate and output voltage measured by the M-TENG with respiration was investigated. A total of 40 respiration rates and output voltages were utilized at each sleep stage to acquire an averaged value (*μ*) and standard deviation (*σ*), respectively. Each value of *μ* and *σ* can be checked, as shown in Table S2. The highest values of the standard deviation in the respiration rate and output voltage were recorded in the REM stage, which is consistent with the results, as shown in Figure 5e,f. Additionally, in contrast to the REM stage, a regular pattern of the respiration was confirmed during non-REM sleep. To confirm the same respiration pattern with participant A, an additional experiment was conducted, as shown in Figure S3. These results show the M-TENG successfully confirmed changes in respiration during sleep.

Although the changes in the respiration of participant A were successfully confirmed, the values of the respiration rate and electrical signal can vary depending on the individual characteristics. Accordingly, the electrical signal generated from the M-TENG and respiration rate of participant B and C were additionally investigated as shown in Figures S4 and S5. The acquired values from participants B and C showed similar patterns according to sleep stage (Tables S3 and S4). The respiration rates and output voltages acquired from participants A, B, and C were compared, as shown in Figure S6. Although the generated signals by participants A, B, and C were different, their respiration rates and output voltages showed the same tendency according to sleep stages. The observed differences in the respiration patterns were influenced by the physical characteristics of participants A, B, and C, such as the size of the lungs or nasal structure. Considering these results, the sleep stage can be detected by confirming the respiration rate and output voltage acquired by the M-TENG at each sleep stage.

### 3.3. Data Process of the Proposed System with the K-Mean Clustering and Classification Results

**Figure 6:**
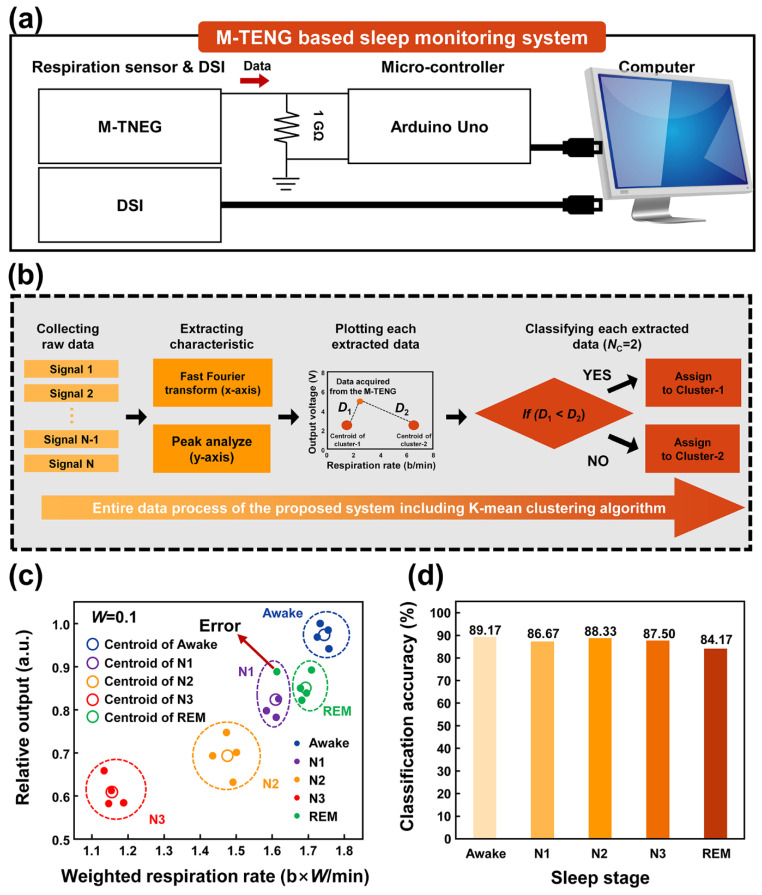
The overall process of the pattern classification with electrical signal generated from the M-TENG and its classification accuracy. (**a**) Schematic diagram describing the proposed system and (**b**) k-mean clustering-based data process for pattern classification. (**c**) The plotted data and its classification results using the featured data. (**d**) The classification accuracy of the sleep stages according to arbitrary respiration.

The potential utilization of the M-TENG for monitoring the sleep stages was successfully confirmed because the electrical signals generated from the M-TENG showed the specific trends for each sleep stage. However, the observed patterns were manually sorted. These manual alignments are a burden, because the sleep monitoring needs to be conducted for a long period of time. This issue can be easily solved with the aid of the machine learning approach, because the common features in each arbitrary data can be detected and analyzed by the machine learning approach. To verify the sleep stage using the M-TENG, a data process was designed and implemented, as shown in Figure 6a. The measurement was conducted by measuring the electrical signal generated from the M-TENG and DSI at the same time. Each signal was transmitted through the wire, as shown in Figure 6a. The raw data of the respiration patterns generated from the M-TENG were measured by connecting a load resistance of 1 GΩ to an Arduino board and then transferred to the computer through the Arduino board. With a large load resistance, the electrical signal generated from the M-TENG can be transferred to the computer, with only a small loss. Additionally, due to the external power source of the Arduino board, noise was created. To suppress this noise, a noise removing filter was designed, which can remove the offset generated from the Arduino board due to the external power source. The classification process for the acquired data was based on the k-mean clustering method, as shown in Figure 6b. To classify the sleep stage, raw data was first collected with the M-TENG. The respiration rate and output voltage were extracted based on the FFT and peak analyzing algorithm, respectively. The extracted respiration rate and output voltage were allocated as the *x*-axis and *y*-axis, respectively. Then, the distances between the plotted data and centroids of each cluster were calculated. The distance between cluster-1 and plotted data was defined as the *D*_1_ and the distance between cluster-*i* and plotted data was defined as *D_i_*. If the *D*_1_ is shorter than the *D_i_*, the plotted data is allocated to cluster-1. The distance between centroid A and generated points can be calculated via the following Equation (1):

(1) $$D_{i}=\sqrt{{\left(x_{i}-a\right)}^{2}+{\left(y_{i}-b\right)}^{2}}$$

where *i* is the number of the clusters when the order pair of the centroids is (*x_i_*, *y_i_*), and *a* and *b* are data of the participant whose ordered pair is (*a*, *b*).

If the *D*_1_ is smaller than *D*_2_, the data acquired from the M-TENG should be assigned to cluster-1 because this result indicates that the data acquired from the M-TENG possesses the similar output voltage and respiration rate with cluster-1 than cluster-2. For example, if the M-TENG generates the 3.5 V with a respiration rate of 17.5, the generated data from these can be expressed as (17.5, 3.5). The centroid of A and B are (17.5, 0) and (13.5, 1), respectively. Then, the distance between centroid A and the data of the participant can be calculated as 3.5. On the other hand, the distance between centroid B and the data of person B can be calculated as 4.72. Therefore, the data of the participant is included in the cluster, which possesses centroid A. Because there are five sleep stages, the number of clusters (*N*_C_) was five in this study. The entire flow chart of the k-mean clustering-based classification model can be checked in Figure S7.

With the proposed data process, centroids possessing the feature of each sleep stage were successfully created as shown in Figure 6c. The small circles indicate the data confirming the sleep stage through the EEG analysis. The circles possess the unique characteristics of each sleep stage. The hollow circles with dotted lines show the sleep stage classified using the proposed data process. Because the unstable respiration pattern occurred during the REM stage, classification error occurred in the REM sleep. Nevertheless, the sleep stage was well distinguished with the proposed data process. However, this classification was conducted based on the data possessing the unique characteristics of each sleep stage. Because the changes in the sleep stage were not digitally performed, for the actual sleep monitoring the classification of the sleep stage should be conducted using arbitrary data. The data acquired by participants was randomly utilized to classify the sleep stage. The classification accuracy recorded for the REM stage was the lowest due to its unstable characteristics. However, the proposed data process showed high classification accuracy of 87.17% by the M-TENG with the aid of the k-mean clustering-based algorithm, as shown in Figure 6d. These results indicate that the proposed combination can improve self-awareness of sleep disorders by classifying sleep stages with the high average classification accuracy. Additionally, the verification was conducted with five different algorithms including k-mean clustering, naive bayes, logistic regression, decision tree, and support vector machine. In each sleep stage, other algorithms showed higher classification accuracy, but the k-mean clustering algorithm showed the highest averaged classification accuracy among them, as shown in Figure S8. Considering these results, the proposed combination of the M-TENG and machine learning approach is expected to be utilized in the design of smart sleep monitoring systems in near future.

## 4. Discussion

### 4.1. Effect of Humidity in the Electrical Output Generated from the M-TENG

**Figure 7:**
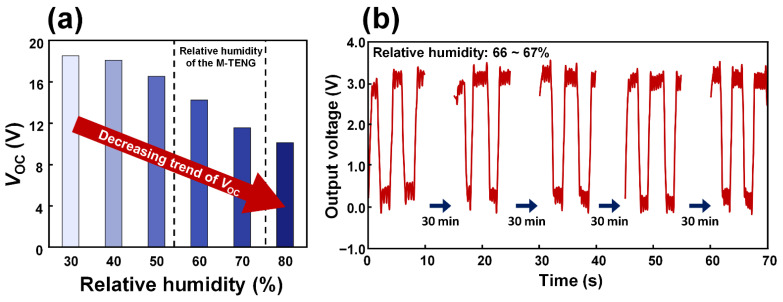
The electrical output generated from the M-TENG according to the relative humidity. (**a**) The *V*_OC_ measured at relative humidity within 30 to 80%. (**b**) The *V*_OC_ measured at relative humidity of 66 to 67% for 2 h.

The relative humidity can affect the electrical output generated from the M-TENG. To confirm the effect of the relative humidity in the electrical output generated from the M-TENG, the *V*_OC_ of the M-TENG was investigated according to the relative humidity. As a result, the electrical output generated from the M-TENG decreased as relative humidity increased, as shown in Figure 7a. However, the relative humidity in the M-TENG was saturated at 66 to 67%. With this relative humidity, the additional experiment was conducted to demonstrate the consistent signal generated from the M-TENG with breathing. For 2 h, the electrical outputs generated from the M-TENG were investigated with the interval for 30 min. Then, the electrical outputs generated from the M-TENG with breathing were nearly constant, as shown in Figure 7b.

### 4.2. Effect of the Tilted RIE Process into the Electrical Output Generated from the M-TENG

**Figure 8:**
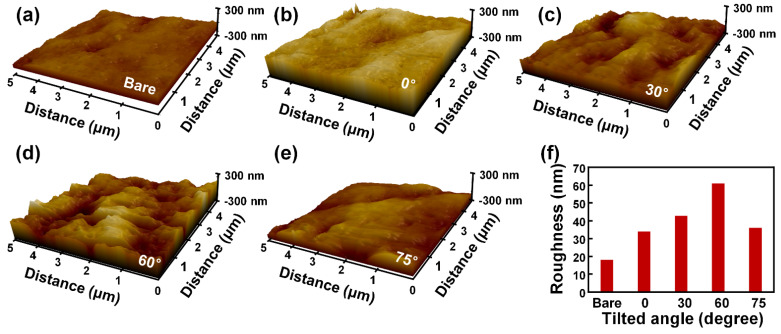
Effect of the tilted RIE process into electrical output of the M-TENG. Surface morphology of the (**a**) bare PTFE film, PTFE film etched with tilted angle of (**b**) 0°, (**c**) 30°, (**d**) 60°, and (**e**) 75°. (**f**) The surface roughness of each PTFE film.

In Figure 8, the surface of the PTFE films was investigated through atomic force microscopy (AFM) images. A pressure sensitivity of the M-TENG is crucial to detect the respiration because the weak air pressure is produced by respiration. By increasing the electrical output generated from the M-TENG with the same air-induced pressure, the pressure sensitivity of the M-TENG can be enhanced. The electrical output generated from the TENG can be enhanced by increasing effective contact area and surface charge density. To increase the pressure sensitivity, the RIE process was conducted with different tilted angles to form the different surface morphology on the PTFE film. Figure 8 showed each surface morphology and roughness value of the PTFE films after the RIE process with different titled angles for etching. To implement different tilting angles, structures with different angles were fabricated and PTFE films were attached on the structures. Compared to the bare PTFE film (Figure 8a), the roughness values of the PTFE film were increased after tilted RIE process (Figure 8b–e). These changes can be confirmed at Figure 8f.

### 4.3. Optimizing the Parameter for Increasing Classification Accuracy of the Proposed System

**Figure 9:**
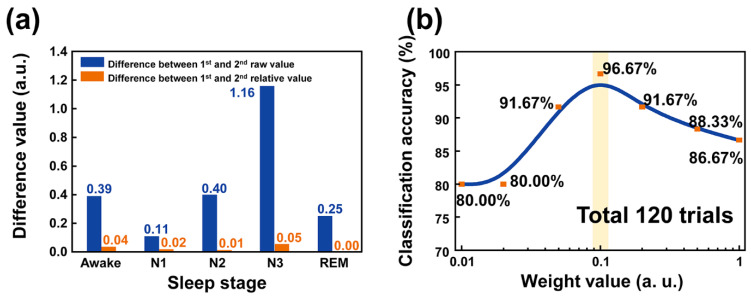
(**a**) The difference value for each stage during the sleep, comparing the difference between raw data and relative value. (**b**) The classification accuracy of the sleep stages according to the weight value for respiration rate.

Respiration during sleep can be changed due to the condition of the body. This change in respiration can affect the classification results because classifying the sleep stage is based on the distance between each data point acquired by the M-TENG. To suppress this error, a relative value was adopted. The relative value was calculated by dividing the highest amplitude of the output voltage among the raw data. The differences between raw data and relative value were investigated, as shown in Figure 9a. For Figure 9a, the electrical output as shown in Figure S3b was utilized. Since the electrical output measured at the awake state on day 2 was the highest value among the electrical outputs generated from the M-TENG, the measured electrical outputs were divided by that value. As a result, the difference between electrical outputs was decreased. As shown in Figure 9a, the relative value shows a smaller difference value than the raw data. To calculate the relative value in this manuscript, the highest electrical output was selected as the standard. Considering these results, the relative value was adopted to classify the sleep stage with the M-TENG.

With the k-mean clustering-based algorithm, the centroid is regarded as the most important factor to acquire good classification results. This is because the centroid is the standard for classifying the data by calculating the distance of each arbitrary data point. With the relative output voltage, the classification was conducted. As a result, the classification accuracy of 86.67% was recorded with the data including the characteristics of each sleep stage. However, when the weight value (W) was multiplied to the respiration rate, the classification accuracy was changed. For example, for weight values from 1 to 0.1, the classification accuracy was increased to 86.67%, 88.33%, 91.67%, and 96.67%, respectively. On the other hand, the classification accuracies were 96.67%, 91.67%, 80.00%, and 80.00%, when the weight values were 0.1, 0.05, 0.02, and 0.01, respectively, as shown in Figure 9b. Before multiplying the weight value to the respiration rate, the distance between the data point and each centroid mainly depends on the respiration rate. This is because the value of the respiration rate is higher than that of the relative output by at least 10 times. Hence, the effect of the relative output into calculating the distance between the centroid of the cluster and the data point was smaller than that of the respiration rate into the distance. By multiplying weight value to the respiration rate, the difference in value between the relative output and respiration rate can be reduced, and this reduction can suppress classification errors. As a result, when the value of the respiration rate became similar to that of the relative output voltage by multiplying the weight value, the highest classification accuracy of 96.67% was recorded, with the optimal weight value of 0.1. This is because the values of the x-axis and y-axis are altered to a similar order by multiplying the weight value of 0.1 to the respiration rate. Then, when the sleep stage was classified based on the calculated distance, the values of the x-axis and y-axis with the similar order affected the distance between the data point and centroid of the cluster at the same time. On the other hand, when the weight of the respiration rate was smaller than 0.1, the effect of the weighted respiration rate, which was allocated at the x-axis, into determining the distance between centroids of the cluster and itself was gradually decreased. This reduced effect of the weighted respiration rate on the classification accuracy can be observed at the weight values of 0.05, 0.02, and 0.01. In particular, when the weight value was smaller than 0.05, the classification accuracy was saturated at 80.00%. This result indicates that the effect of the weighted respiration rate no longer had an effect on the distance between the centroid and data. When weight values less than 0.05 were applied to the respiration rate, the distance between the centroid of the cluster and data was predominantly determined by the relative output. Then, the weight value of 0.1 was adopted for the respiration rate.

## 5. Conclusions

In summary, five sleep stages could be classified using the combination of the mask-shaped triboelectric nanogenerator (M-TENG) and machine learning approach. The fabricated M-TENG consisted of an Al electrode, PTFE film, and ABS-A100 frame. The weight of the attached PTFE film was optimized to accurately detect respiration. The fabricated M-TENG demonstrated its excellent potential as a respiration sensor because shallow, normal, and deep breathing through nose and mouth were successfully detected with the M-TENG. Additionally, the respiration pattern was investigated as the sleep stage changed. As a result, the relationship between respiration patterns and sleep was successfully detected. Based on this result, the M-TENG was utilized to classify the sleep stages with the aid of a k-mean clustering-based data process. Relative output voltage was adopted to increase the classification accuracy for each sleep stage. The weight value of the respiration rate was also optimized and systematically analyzed. As a result, the highest classification accuracy of 96.67% was recorded at the weight value of 0.1 with the featured data of each sleep stage. Finally, each sleep stage was classified using arbitrary data. Classification accuracies of 89.17%, 86.67%, 88.33%, 87.50%, and 84.17% were recorded for sleep stages of awake, N1, N2, N3, and REM, respectively. Finally, the averaged classification accuracy of 87.17% was recorded indicating that the proposed combination can be used to help recognize various sleep disorders, based on the classified sleep stages with the high classification accuracy. Considering these results, the proposed combination of the M-TENG and machine learning approach for sleep monitoring is expected to be utilized as a promising smart sleep monitoring system in the near future.

## Data Availability

Data sharing is not applicable to this article.

## Supplementary Materials

The following supporting information can be downloaded at: https://www.mdpi.com/article/10.3390/polym14173549/s1, Figure S1: The electrical output generated from the M-TENG with various weights of the PTFE film. (a) The open-circuit voltage with various weights of the PTFE. (b) The short-circuit current with various weights of the PTFE. Figure S2: Actual image of the DSI-24 and its electrode locations. Figure S3: The electrical signal generated from the M-TENG for participant A on the 1st day and 2nd day, respectively. (a) The respiration rates of participant A on the 1st and the 2nd days. (b) The output voltage of participant A on the 1st and 2nd day. Figure S4: The distribution of the respiration rate and output voltage generated from the M-TENG by the respiration of participant B with the total of 40 data. (a) The distribution of the respiration rates conducted by participant B. (b) The distribution of the output voltage generated from the M-TENG by the respiration of participant B. Figure S5: The distribution of the respiration rate and output voltage generated from the M-TENG by the respiration of participant C with the total of 40 data. (a) The distribution of the respiration rates conducted by participant C. (b) The distribution of the output voltage generated from the M-TENG by the respiration of participant B. Figure S6: Comparison of the respiration rate and output voltage generated from the M-TENG by the respiration of the participants. (a) A comparison of the respiration rates of participants A, B, and C. (b) A comparison of the output voltage generated from the M-TENG by the respiration of participants A, B, and C. Figure S7: Flow chart of the k-mean clustering-based classification model. Figure S8: Comparison of the classification accuracy with five different algorithms. Table S1: Output voltage values according to the sleep stage with participant A. Table S2: Mean values of electrical output and respiration rate and their values of standard deviation for participant A according to sleep stage. Table S3: Mean values of electrical output and respiration rate and their values of standard deviation for participant B according to sleep stage. Table S4: Mean values of electrical output and respiration rate and their values of standard deviation for participant C according to sleep stage.
