# Supplementary material for: A Mask-Shaped Respiration Sensor Using Triboelectricity and a Machine Learning Approach toward Smart Sleep Monitoring Systems
Source: Polymers (Basel). 2022 Aug 29;14(17):3549. doi: 10.3390/polym14173549 (PMC9460850; doi:10.3390/polym14173549)
Supplement: Supplementary file 1 [file polymers-14-03549-s001.zip › polymers-1855283-SI.pdf]

Supporting Information

A Mask-Shaped Respiration Sensor Using Triboelectricity and a Machine Learning Approach toward Smart Sleep Monitoring Systems

Jonghyeon Yun[1,3], Jihyeon Park[1,3], Suna Jeong[4], Deokgi Hong[4,], and Daewon Kim[2,*]*

[1] Department of Electronics and Information Convergence Engineering, Institute for Wearable Convergence Electronics, Kyung Hee University, 1732 Deogyeong-daero, Giheung-gu, 17104 Yongin, Korea

[2] Department of Electronics and Information Convergence Engineering, Institute for Wearable Convergence Electronics, Kyung Hee University, 1732 Deogyeong-daero, Giheung-gu, 17104 Yongin, Korea

[3] Institute for Wearable Convergence Electronics, Kyung Hee University, 1732 Deogyeong-daero, Gi-heung-gu, 17104 Yongin, Korea

[4] Department of Occupational Therapy, College of Medicine, Wonkwang University, Institution of Envi-roscience, 460 Iksan-daero, Jeollabuk-do, 54538 Iksan, Korea

* Corresponding author

Email address: hongdgot84@wku.ac.kr (D. G. Hong); daewon@khu.ac.kr (D. Kim)

Table of contents

1. The electrical output generated from the M-TENG with various weights of the PTFE film.
2. Actual image of the DSI-24 and electrode locations of the DSI-24.
3. The electrical signal generated from the M-TENG for participant A on the 1st day and 2nd day.
4. The distribution of the respiration rate and output voltage generated from the M-TENG by the respiration of the participant B with the total of 40 data.
5. The distribution of the respiration rate and output voltage generated from the M-TENG by the respiration of the participant C with the total of 40 data.
6. Comparison of the respiration rate and output voltage generated from the M-TENG by the respiration of participants A, B, and C.
7. Flow chart of the k-mean clustering-based classification model
8. Comparison of the classification accuracy with five different algorithms.
9. Output voltage values according to the sleep stage with participant A.
10. Mean values of voltage and respiration rate and their values of standard deviation for participant A according to sleep stage.
11. Mean values of voltage and respiration rate and their values of standard deviation for participant B according to sleep stage.
12. Mean values of voltage and respiration rate and their values of standard deviation for participant C according to sleep stage.

1. The electrical output generated from the M-TENG with various weights of the PTFE film.

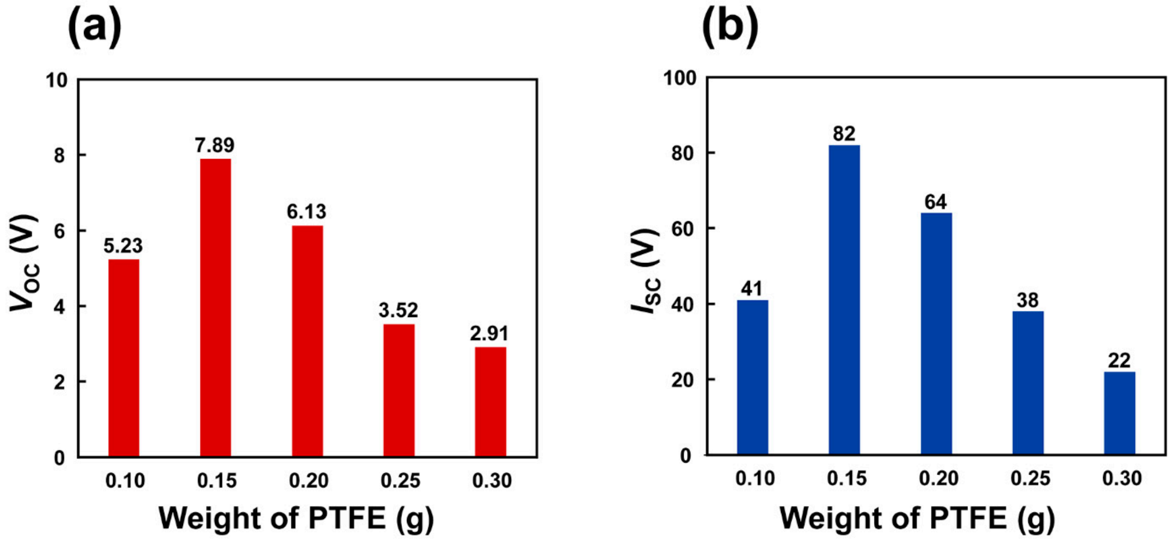

Figure S1. The electrical output generated from the M-TENG with various weights of the PTFE film. (a) The open-circuit voltage with various weights of the PTFE. (b) The short-circuit current with various weights of the PTFE.

2. Actual image of the DSI-24 and electrode locations of the DSI-24.

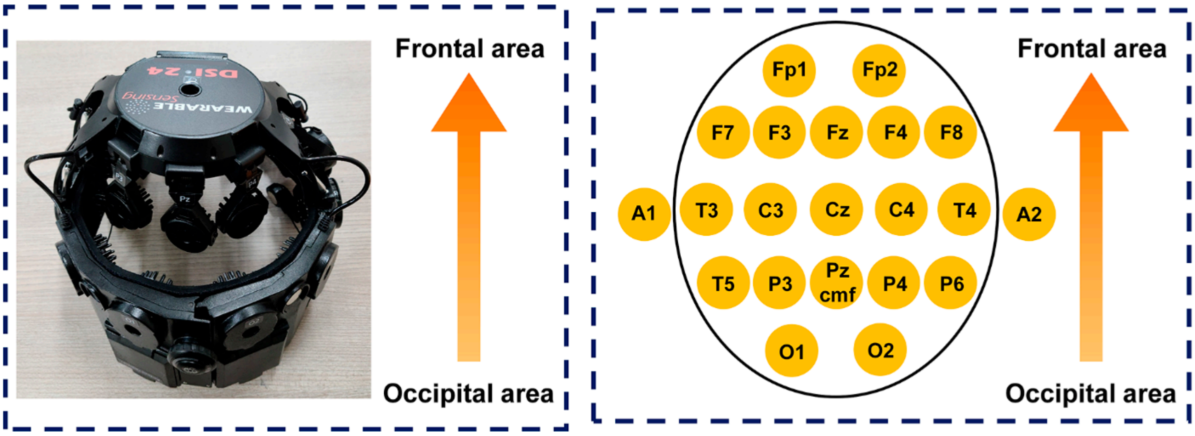

Figure S2. Actual image of the DSI-24 and its electrode locations.

3. The electrical signal generated from the M-TENG for participant A on the 1st day and 2nd day.

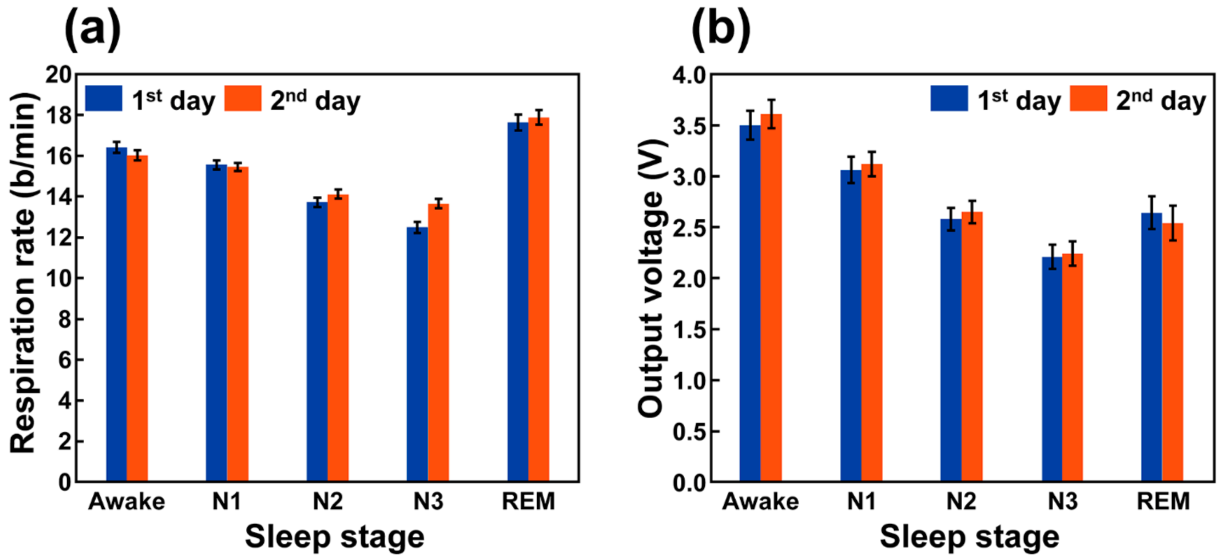

Figure S3. The electrical signal generated from the M-TENG for participant A on the 1st day and 2nd day, respectively. (a) The respiration rates of participant A in 1st day and 2nd. (b) The output voltage of participant A on the 1st day and 2nd.

4. The distribution of the respiration rate and output voltage generated from the M-TENG by the respiration of the participant B with the total of 40 data.

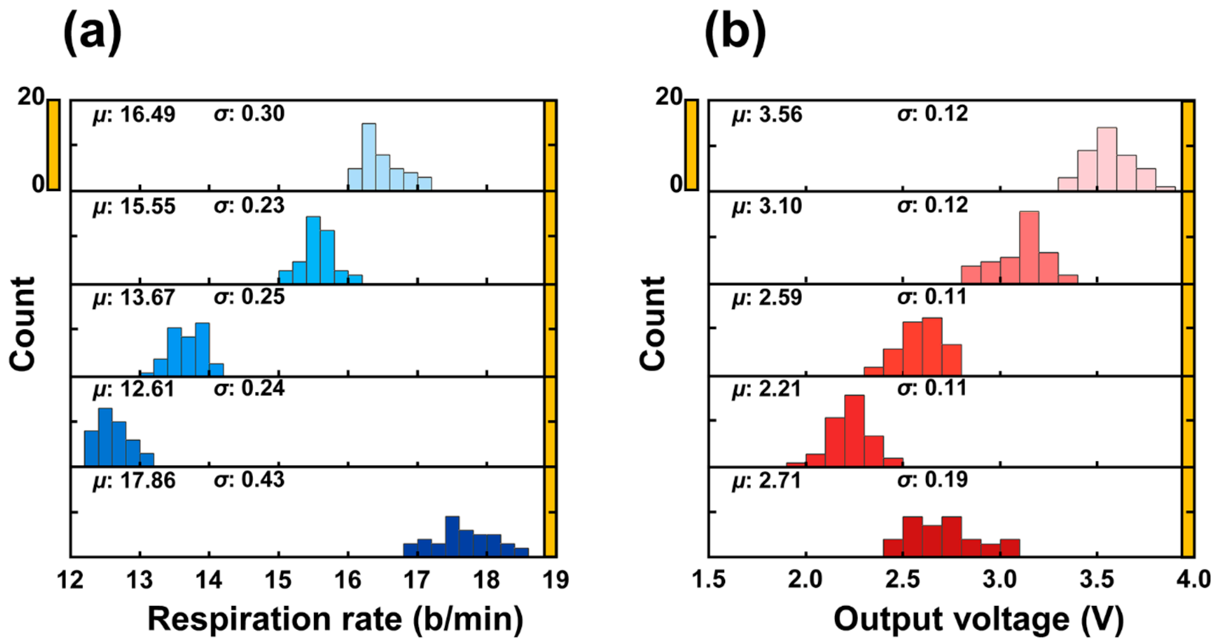

Figure S4. The distribution of the respiration rate and output voltage generated from the M-TENG by the respiration of the participant B with the total of 40 data. (a) The distribution of the respiration rates conducted by participant B. (b) The distribution of the output voltage generated from the M-TENG by the respiration of the participant B.

5. The distribution of the respiration rate and output voltage generated from the M-TENG by the respiration of the participant C with the total of 40 data.

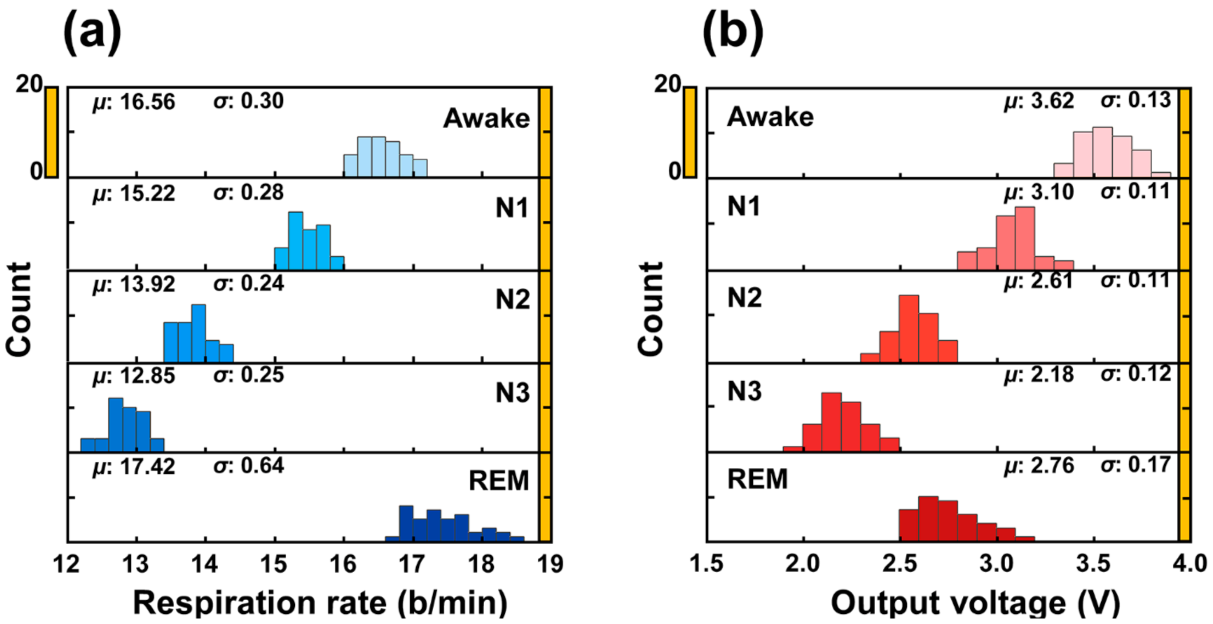

Figure S5. The distribution of the respiration rate and output voltage generated from the M-TENG by the respiration of the participant C with the total of 40 data. (a) The distribution of the respiration rates conducted by participant C. (b) The distribution of the output voltage generated from the M-TENG by the respiration of the participant B.

6. Comparison of the respiration rate and output voltage generated from the M-TENG by the respiration of participants A, B, and C.

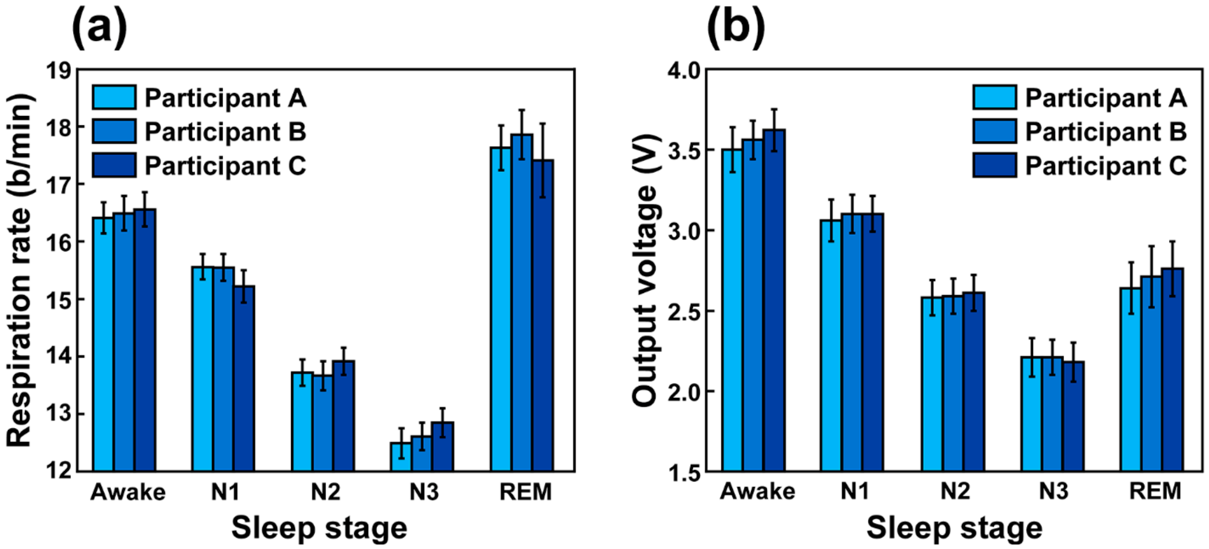

Figure S6. Comparison of the respiration rate and output voltage generated from the M-TENG by the respiration of the participants. (a) The comparison of the respiration rates of participants A, B, and C. (b) The comparison of the output voltage generated from the M-TENG by the respiration of participants A, B, and C.

7. Flow chart of the k-mean clustering-based classification model.

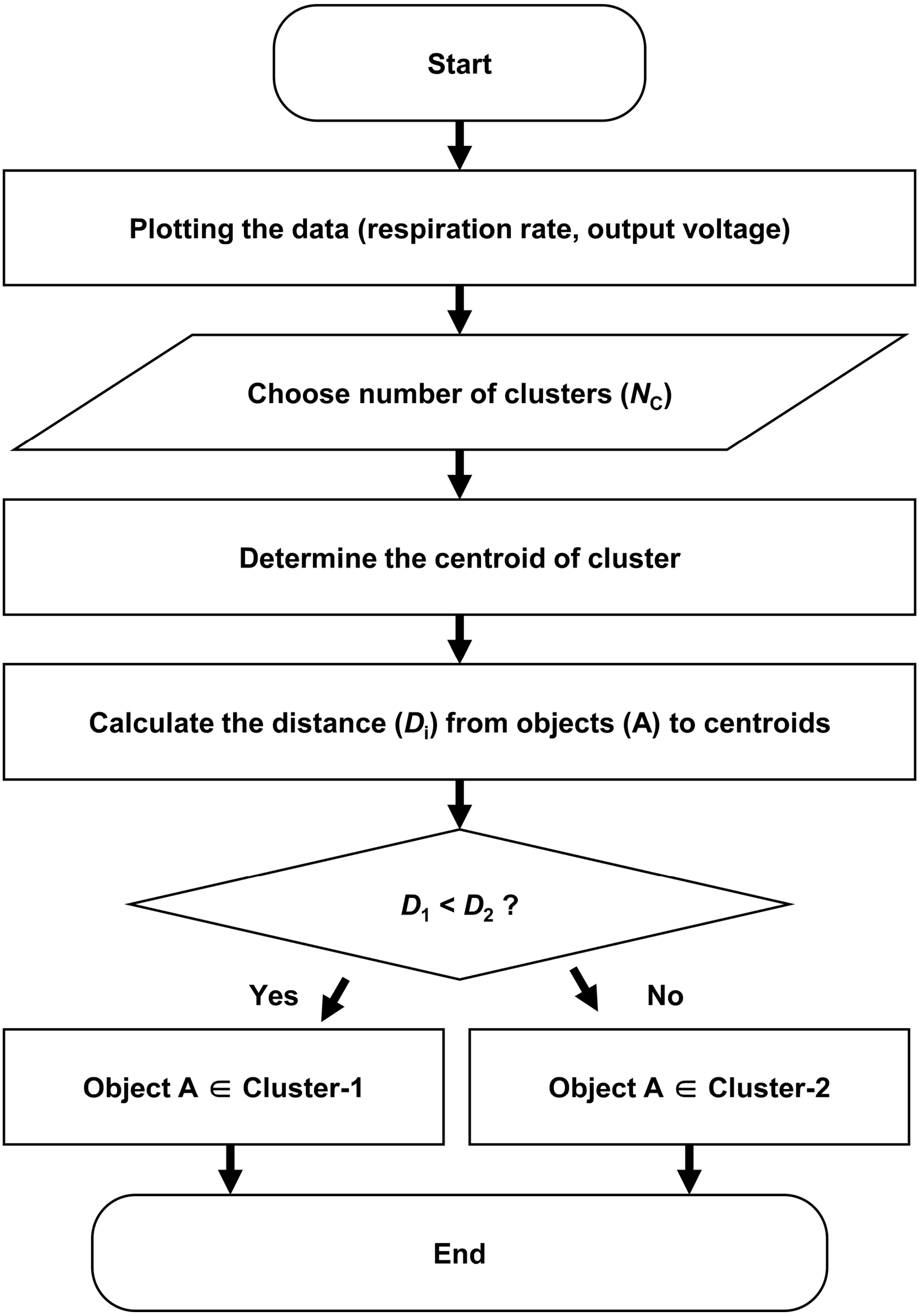

Figure S7. Entire flow chart of the k-mean clustering-based classification model.

8. Comparison of the classification accuracy with five different algorithms.

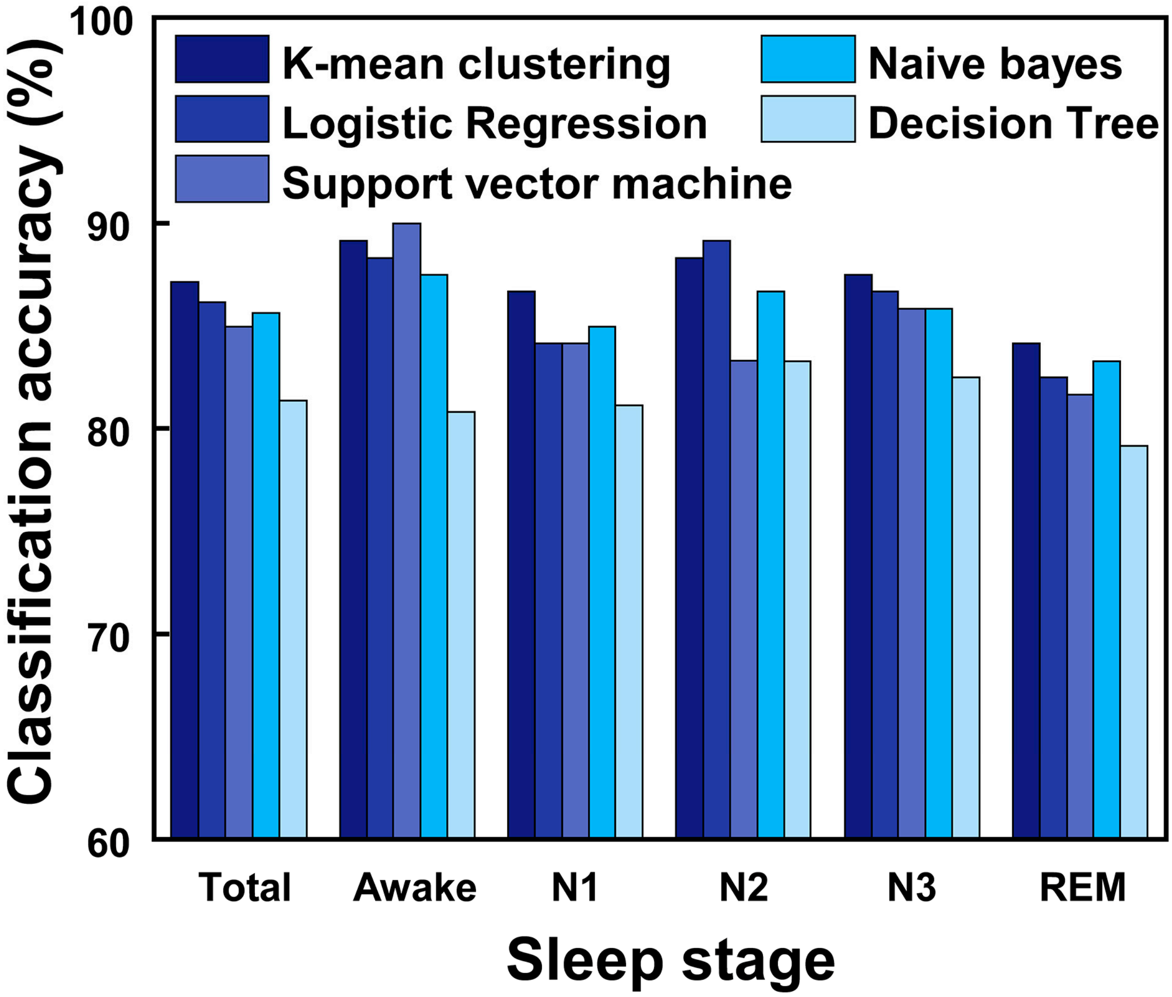

Figure S8. Comparison of the classification accuracy with five different algorithms.

9. Output voltage values according to the sleep stage with participant A

Sleep stage	Awake	N1	N2	N3	REM
**Mean value of the voltage (V)**	**3.562**	**3.084**	**2.655**	**2.312**	**2.612**

Table S1. Measured output voltage according to the sleep stage with participant A

10. Mean values of voltage and respiration rate and their values of standard deviation for participant A according to sleep stage.

Sleep stage	Awake	N1	N2	N3	REM
**Mean value of the voltage (V)**	**3.50**	**3.06**	**2.58**	**2.21**	**2.64**
**standard deviation of voltage**	**0.14**	**0.13**	**0.11**	**0.12**	**0.16**
**Mean value of respiration rate (b/min)**	**16.41**	**15.56**	**13.72**	**12.49**	**17.63**
**standard deviation of respiration rate**	**0.27**	**0.22**	**0.23**	**0.26**	**0.39**

Table S2. Mean values of electrical output and respiration rate and their values of standard deviation for participant B according to sleep stage.

11. Mean values of voltage and respiration rate and their values of standard deviation for participant B according to sleep stage.

Sleep stage	Awake	N1	N2	N3	REM
**Mean value of the voltage (V)**	**3.56**	**3.10**	**2.59**	**2.21**	**2.71**
**standard deviation of voltage**	**0.30**	**0.23**	**0.25**	**0.24**	**0.43**
**Mean value of respiration rate (b/min)**	**16.49**	**15.55**	**13.67**	**12.61**	**17.86**
**standard deviation of respiration rate**	**0.12**	**0.12**	**0.11**	**0.11**	**0.19**

Table S3. Mean values of electrical output and respiration rate and their values of standard deviation for participant B according to sleep stage.

12. Mean values of voltage and respiration rate and their values of standard deviation for participant C according to sleep stage.

Sleep stage	Awake	N1	N2	N3	REM
**Mean value of the voltage**	**3.62**	**3.10**	**2.61**	**2.18**	**2.76**
**standard deviation of voltage**	**0.30**	**0.28**	**0.24**	**0.25**	**0.64**
**Mean value of respiration rate**	**16.56**	**15.22**	**13.92**	**12.85**	**17.42**
**standard deviation of respiration rate**	**0.13**	**0.11**	**0.11**	**0.12**	**0.17**

Table S4. Mean values of electrical output and respiration rate and their values of standard deviation for participant C according to sleep stage.
